# The multifactorial nature of beak and skull shape evolution in parrots and cockatoos (Psittaciformes)

**DOI:** 10.1186/s12862-019-1432-1

**Published:** 2019-05-17

**Authors:** Jen A. Bright, Jesús Marugán-Lobón, Emily J. Rayfield, Samuel N. Cobb

**Affiliations:** 1 School of Geosciences, University of South Florida, Tampa, FL 33620 USA; 2 Unidad de Paleontologıa, Departamento Biologıa, Universidad Autonoma de Madrid, Cantoblanco, 28049 Madrid, Spain; 3 Dinosaur Institute, Natural History Musuem of Los Angeles County, Los Angeles, CA 90007 USA; 4 School of Earth Sciences, University of Bristol, Bristol, BS8 1TQ UK; 5 Department of Archaeology, University of York, York, YO10 5DD UK; 6 Hull York Medical School, University of York, York, YO10 5DD UK

**Keywords:** Birds, Geometric morphometrics, Allometry, Integration, Feeding, Parrots

## Abstract

**Background:**

The Psittaciformes (parrots and cockatoos) are characterised by their large beaks, and are renowned for their ability to produce high bite forces. These birds also possess a suite of modifications to their cranial architecture interpreted to be adaptations for feeding on mechanically resistant foods, yet the relationship between cranial morphology and diet has never been explicitly tested. Here, we provide a three-dimensional geometric morphometric analysis of the developmental and biomechanical factors that may be influencing the evolution of psittaciformes’ distinctive cranial morphologies.

**Results:**

Contrary to our own predictions, we find that dietary preferences for more- or less- mechanically resistant foods have very little influence on beak and skull shape, and that diet predicts only 2.4% of the shape variation in psittaciform beaks and skulls. Conversely, evolutionary allometry and integration together predict almost half the observed shape variation, with phylogeny remaining an important factor in shape identity throughout our analyses, particularly in separating cockatoos (Cacatuoidea) from the true parrots (Psittacoidea).

**Conclusions:**

Our results are similar to recent findings about the evolutionary trajectories of skull and beak shape in other avian families. We therefore propose that allometry and integration are important factors causing canalization of the avian head, and while diet clearly has an influence on beak shape between families, this may not be as important at driving evolvability within families as is commonly assumed.

**Electronic supplementary material:**

The online version of this article (10.1186/s12862-019-1432-1) contains supplementary material, which is available to authorized users.

## Background

The overwhelming variety of forms presented by the skulls of modern birds have long been a source of inspiration for evolutionary and functional morphologists. Much of the skull disparity among the 11,000 or so species of extant birds is manifested in the shape of the beak, and there is a demonstrable link between the shape of this structure and preferred diet [1, 2]. However, the link between beak shape and diet, or feeding behaviour, is far from simple [3–5], and a myriad of other behavioural [6–8], homeostatic [9], evolutionary [3, 10–13], and structural and developmental factors [14, 15] work to generate variation in form in the beak and the rest of the skull.

In a previous paper, we showed that allometry, the concomitant change of shape with size, coupled to a strong integration between the beak and braincase, can account for some 80% of the phenotypic variation in raptorial birds [3]. As raptors are a polyphyletic group made up of several early-branching non-passerine landbird clades [16, 17], we suggested that integration underpinned by allometry may be the basal phenotypic condition organising the skulls of landbirds, a large and disparate clade comprising approximately 75% of all neoavian species. If true, such integration and allometry could highly constrain the range of shapes that may evolve within landbird families, and variation in the strength of this signal may have been critical in facilitating the propensity to vary (i.e., evolvability).

The parrots and cockatoos (Order: Psittaciformes) are a distinguishable group of landbirds sister to the passerines, and with 398 currently recognised species, they are one of the largest non-passerine clades [18]. These gregarious and intelligent birds are characterised by their brightly-coloured plumage and the presence of a deep, broad, and highly curved beak [18]. This beak, together with a muscular tongue and a suite of musculoskeletal adaptations affecting the palate, craniofacial hinge, and jaw adductors [19, 20] is used for extensive oral pre-processing of vegetation during feeding, and many Psittaciformes are anecdotally renowned for their ability to produce considerable bite forces in order to feed upon hard and tough nuts and seeds [18, 21, 22]. Furthermore, body mass within the group spans several orders of magnitude (from 12.1 g [*Micropsitta*] to 1331 g [*Anodorhynchus*] in flighted species, up to 2000 g in the flightless kakapo [*Strigops*] [23]). Psittaciformes’ beaks are therefore likely subject to high selective pressures to accommodate high feeding forces, and thus provide an ideal test of the prevalence of allometry and integration in non-passerine landbirds where one might expect a strong functional signal.

Here, we use 3D geometric morphometrics to determine the extent to which allometry and integration are present in psittaciform skulls and beaks, and quantify the amount of variation that can be attributed to these factors. Based on our previous results in raptors [3], we hypothesise that both will be significant and major sources of phenotypic variation within the group. We further hypothesise that birds with a preference for diets comprising more resistant foods will have beak and skull shapes that are significantly different to those who prefer softer foods, and that the resistant food feeders will have beaks that are deeper and wider, in order to accommodate higher feeding stresses [2, 24, 25].

## Results

**Fig. 1:**
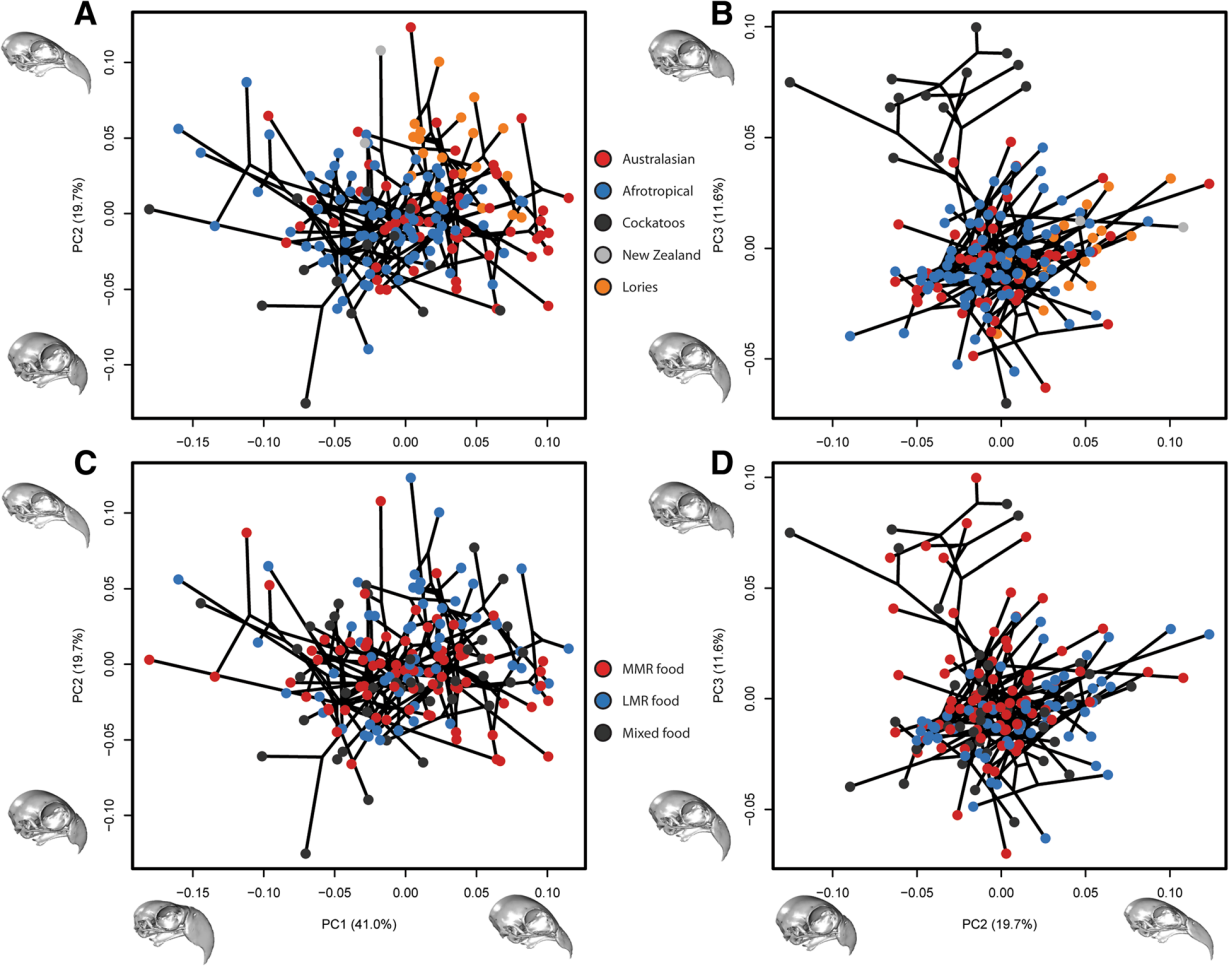
Phylomorphospaces of the original shape data. Points coloured by clade (**a**, **b**) and diet (**c**, **d**). PC1 vs PC2 (**a**, **c**) and PC2 vs PC3 (**b**, **d**). Inset skulls characterise the shapes represented by the maximum and minimum end warps of their corresponding PC axes

Principal Component 1 (PC1; animation in Additional file 1) describes 41.0% of the variation (Fig. 1). Negative scores are associated with inflation of the beak dorsoventrally and anteroposteriorly, but not mediolaterally. There is a corresponding flattening of the skull roof, lengthening of the jugal bars, repositioning of palate-pterygoid complex to a more ventral position relative to rest of skull, and a slight rotation of occipital from a ventral to a more posterior orientation. Such shapes are characteristic of large cockatoos and macaws, whereas positive shapes are exemplified by small parakeets, hanging parrots, lories, and lovebirds. On PC2 (19.7%; animation in Additional file 2), negative scores show a mediolateral widening and dorsoventral deepening of the beak and skull. The beak tip is deflected posteriorly, increasing the notch angle of the tomial edge. The occipital rotates to a more ventral orientation. Negative PC2 scores are characterised by the gang-gang cockatoo (*Callocephalon*) and positive scores are characterised by the vulturine parrot (*Psittrachas*) and the kea (*Nestor notabilis)*. There is a slight tendency for Australasian birds to occupy more positive positions on PCs 1 and 2, whereas cockatoos and Afrotropical birds occupy more negative positions. Positive scores on PC3 (11.6%; animation in Additional file 3) show a dramatic increase in curvature of beak tomial edge, giving the appearance of a sinusoidal curve. A change in the angle between the braincase and the beak gives a concave dorsal profile. Positive PC3 scores are strongly associated with cockatoos, which plot separately to almost all other Psittaciformes, with the notable exception of *Probosciger* (palm cockatoo), which displays an extremely negative PC3 score due to its highly convex facial angle. A significant but small phylogenetic signal (Kmult = 0.344; *p* = 0.001) is evident, also reflected in the significant separation of major clades in PERMANOVA (all pairs *p* = 0.003; *p* = 0.006 if lories designated as separate clade). Psittaciformes with a preference for resistant foods tend to occupy more positive regions of PC3 (Fig. 1d). While PERMANOVA indicates a weakly significant difference between MMR and LMR food feeders (*p* = 0.033; Table S1 in Additional file 4), we made a post-hoc prediction that this trend was driven by the morphological separation of the Cacatuidae (all of whom are MMR or mixed food feeders) from the other Psittaciformes on PC3. A post-hoc PERMANOVA with cockatoos removed showed that birds with different dietary preferences were not significantly different in shape (Table S2 in Additional file 4).

**Fig. 2:**
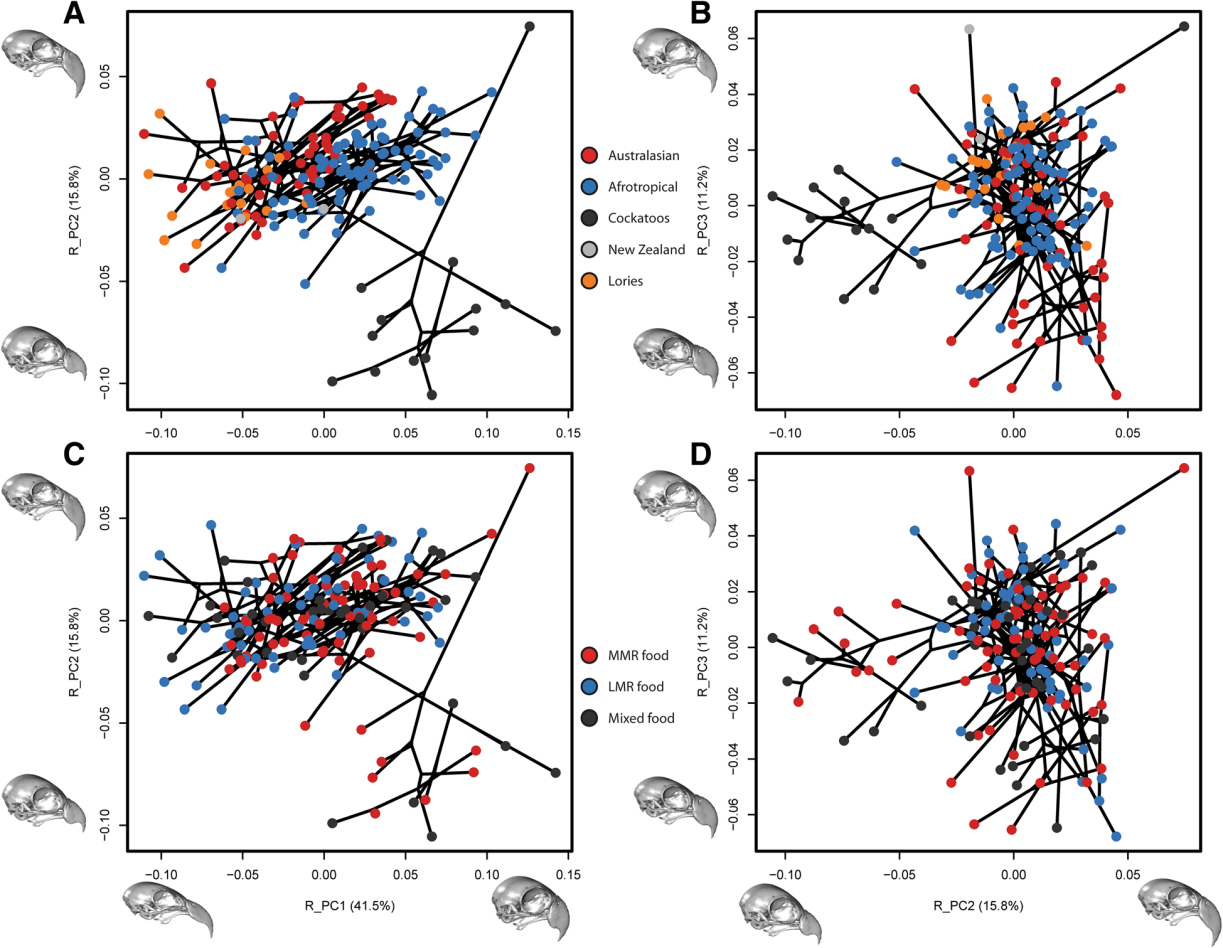
Phylomorphospaces of the residual shapes from the pgls regression of shape and centroid size. Points coloured by clade (**a**, **b**) and diet (**c**, **d**). R_PC1 vs R_PC2 (**a**, **c**) and R_PC2 vs R_PC3 (**b**, **d**). Inset skulls characterise the shapes represented by the maximum and minimum end warps of their corresponding R_PC axes

Linear regressions of shape against centroid size (CS) and diet reveal that both factors are significant (*p* = 0.001), but size predicts over 10 times more shape variation than diet does (CS F = 88.1, R^2^ = 0.338; diet F = 3.11, R^2^ = 0.0239). Much of the allometric variation is associated with phylogeny, as regression using PGLS drops these correlations by approximately half (CS F = 38.3, R^2^ = 0.184, *p* = 0.001; diet F = 1.90, R^2^ = 0.0183, *p* = 0.02). The shape changes predicted by the regression of CS (i.e. allometric shape changes) are very similar to those described by PC1, with larger skulls showing shapes characteristic of negative PC1 scores. Interestingly, the first three PCs of shape variation devoid of allometry (i.e. the PGLS regression residuals: R_PCs 1, 2, and 3; 41.5, 15.8, and 11.2%) are strikingly similar to those of the original PCs 2, 3, and 4 (Fig. 2). Subfamily groups remain distinctive in pairwise comparisons of shape (all pairs *p* = 0.003; *p* = 0.006 if lories designated as separate clade). After accounting for allometry in this way, soft food feeders are significantly different from parrots with MMR (*p* = 0.006) or mixed (*p* = 0.045) food preferences, but only LMR vs MMR food feeders remains significant (*p* = 0.009) when cockatoos are removed (Tables S3, S4 in Additional file 4).

**Fig. 3:**
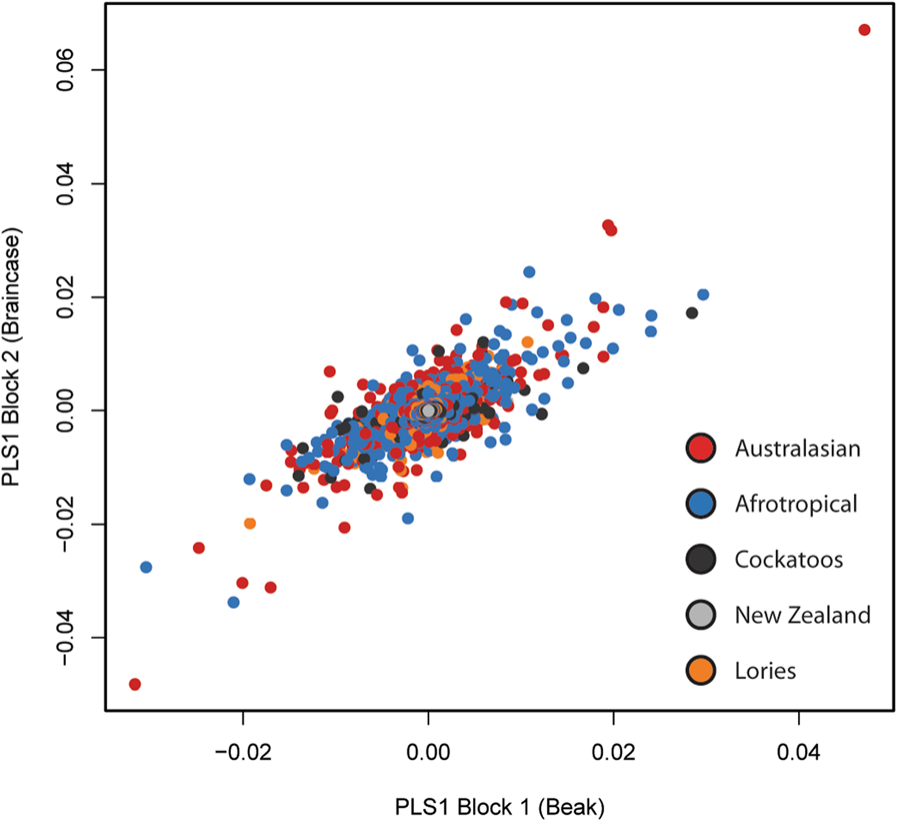
Phylogenetic PLS of the beak and braincase blocks

**Fig. 4:**
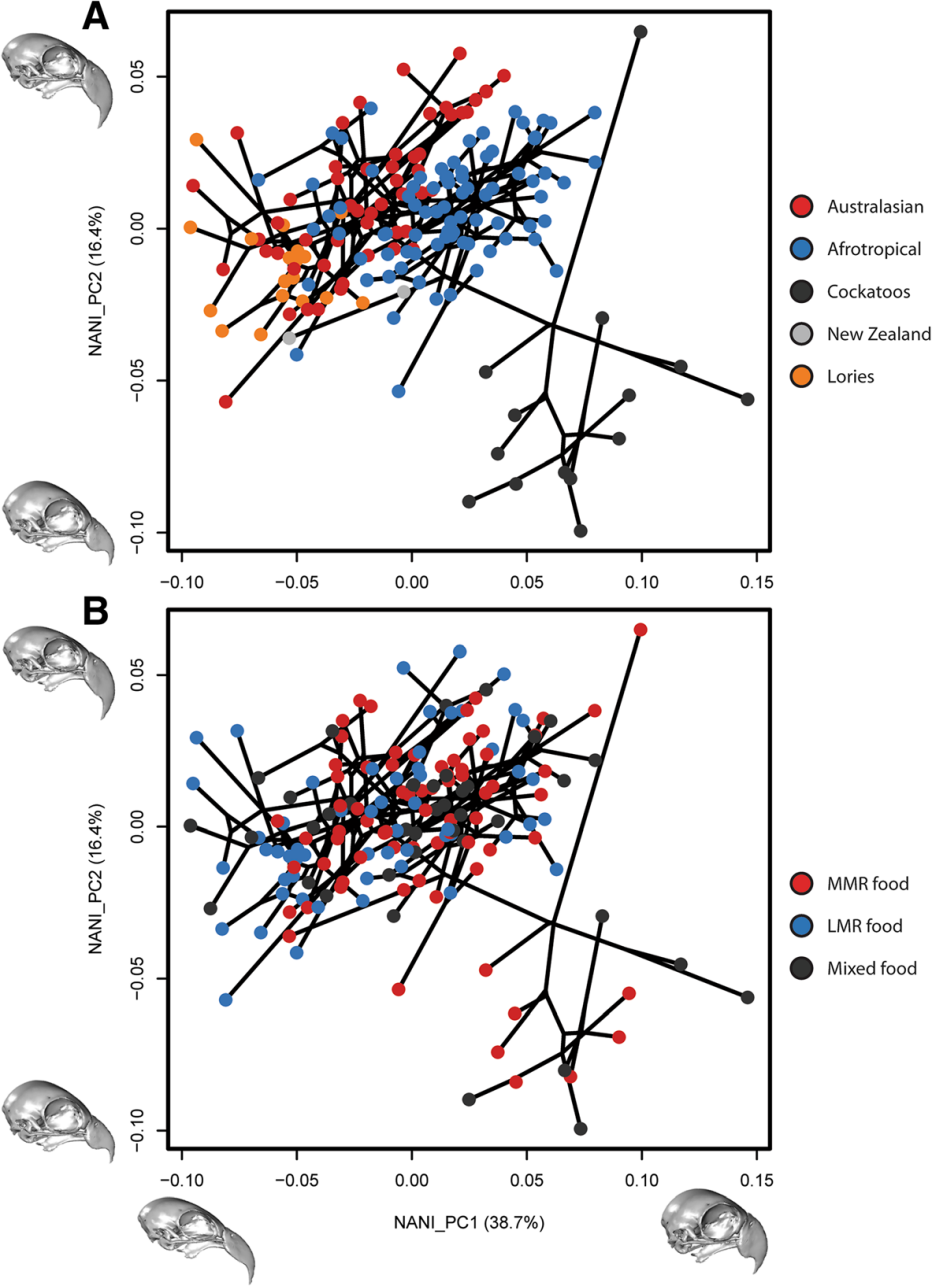
Phylomorphospaces of the NANI (non-allometric, non-integrated) shape data. Points coloured by clade (**a**) and diet (**b**). Inset skulls characterise the shapes represented by the maximum and minimum end warps of their corresponding PC axes

Phylogenetic Partial Least Squares shows that the relationship between beak shape and skull shape is highly significant (rPLS = 0.885, *p* = 0.001; Fig. 3). This relationship between beak and braincase shape remains even after the removal of allometry (rPLS = 0.895, *p* = 0.001). Using the regression procedure outlined in Bright et al. [3], we show that the proportion of skull shape change driven by non-allometric integration is 31.1% (F = 74.8, *p* = 0,001). When considering phylogenetic structure in these regressions, allometry (18.4%) and integration (31.1%) are important factors, together controlling almost half of the skull and beak shape differences in parrots (49.5%). In the remaining 50.5%, phylogeny is significant (Kmult = 0.459, *p* = 0.001) and all subfamily groups are distinctive in pairwise comparisons (all pairs *p* = 0.003; *p* = 0.006 if lories designated as separate clade). Phylogenetic regression of non-allometric non-integrated shape (NANI) to diet shows that diet is not significant (*p* = 1). Plots of the NANI shape in morphospace (Fig. 4) are similar to those of the non-allometric shape (Fig. 2).

## Discussion

In a recent study of the beaks of over 2000 bird genera, Cooney et al. [11] demonstrated that an early burst of phenotypic evolution generated a high disparity of beak shapes between avian clades (niche-expansion), and once these clade-characteristic shapes were established, birds tended to diversify by adjusting their beaks along the same common axes of variation (niche-filling). This observation entails that the major dimensions of shape variation within each avian clade are the same as those within other clades, suggesting that birds have a limited number of means by which to generate beak disparity. This premise of canalization is supported by experimental work showing that bird beaks and skulls develop along constrained genetic pathways [26–29]. Our results also indicate that allometric morphological change and integration between beak and braincase shape variation are two major factors underlying skull structure in psittaciform birds, as they together predict almost half of skull and beak shape. Although we accounted for phylogenetic similarity at every stage of the analyses, the remaining 50.5% of the shape variation appears to be largely associated with phylogenetic inertia, probably driven by the fact that cockatoos have very different skulls and beak shapes to the true parrots. Importantly, our results here echo those of our previous study on birds of prey [3], where allometry and integration predicted 80% of skull shape (admittedly without accounting for the phylogenetic structure, as we have done here).

The confirmed and significant presence of these trajectories in groups as distinctive as the polyphyletic raptors, and now Psittaciformes, clearly emphasises allometry and integration as strong candidates governing the evolutionary pathways of beak shape macroevolution proposed by Cooney et al. [11]. Indeed, integration is often suggested as a mechanism by which evolution may be channelled [30–32], perhaps facilitated by allometry [33]. However, the observation that allometry and integration account for less variation in parrots than in raptors is crucial, as it indicates that while such trajectories may be common in landbirds, they may be of variable importance in the evolvability of morphology among families*.* Parrots and cockatoos famously make extensive mechanical use of their large beaks, but despite a multitude of adaptations to the beak, palate, facial hinge, and jaw musculature, we find that skull and beak shape in these birds is only weakly related to dietary preferences, which account for a mere 2.4% of phenotypic variation. While initially quite surprising, there are several important factors to consider alongside this result. Firstly, while parrots can be broadly classified as seed eaters, and stand apart from other birds in wider morphometric analyses [11], the categories we have used in this study to define the dietary preferences within parrots are necessarily broad. Wild birds are somewhat opportunistic and may experience notable regional or seasonal differences in food availability within their species’ ranges, falling back on generalised herbivory when necessary. Although the fossil details of exactly where and when the crown group Psittaciformes originated remains somewhat ambiguous [34], a major burst of parrot diversification is thought to have occurred during the Miocene, and it has been suggested that this is tied to the aridification of Australia, and the contemporaneous uplift of the Andean and Tibetan plateaus in South America and Asia causing environmental fragmentation [35]. It is possible therefore, that the psittaciform clade is basally adapted to process fall-back foods during times of environmental instability, thus diminishing any signal based on data describing their preferred foods under ideal conditions. Secondly, it is important not to presume that diet is a good proxy for feeding behaviour. Some aspects of function or performance, such as bite force or speed of jaw closure, may demonstrate a tighter correlation to shape. Additionally, the presence of many-to-one mapping patterns between function, form, and performance [36] may allow birds with similarly-shaped beaks to access many different foodstuffs, and for birds with differently-shaped beaks to access the same foodstuff by modifying their behaviour, hence blurring the signal between shape and diet. Thirdly, any morphological study of hard tissues only may under-represent differences in biting performance driven by differences in muscle configuration [37]. This is likely to be an important factor in parrots due to their heavily modified musculature away from the typical avian condition [20, 38], particularly as parrots with a *M. pseudomasseter* and *M. ethmomandibularis* may use these muscles to dramatically improve the efficiency of their jaws by lengthening the in-lever. Lastly, the seed-shelling behaviour of many parrots and cockatoos requires many small, precise, coordinated movements of the jaws and tongue to place and process food within the oral cavity [39]. Some species make notable use of the tongue to place food against small scale ridges and variations in the tomial edge that went unresolved by our landmarking, yet may be associated with different diets, even within the same species [40]. It is therefore possible that more finely resolved dietary categories, improved landmark coverage of more subtle differences in beak shape, or quantification of performance as well as shape, may improve the strength of the dietary signal.

## Conclusions

We conclude that while diet undoubtedly affects the shape of bird beaks, on this phylogenetic scale its effects are overprinted by those of integration and allometry. Our study highlights that, even in structures with an obvious functional role, it is important to explicitly test the influence of a range of evolutionary variables, rather than blindly proceed on the assumption that biomechanical factors are the primary drivers behind organismal form.

## Methods

Bird skulls are hard to scan: the bones are exceptionally thin, even becoming translucent in smaller species, and many useful landmarks are deeply recessed within the highly concave orbits, which are often “shaded” from the scanner. Their small size also makes it exceptionally difficult to manually trace semilandmark curves on physical specimens using a MicroScribe. To compensate for these difficulties without resorting to the prohibitively costly and time-intensive process of X-Ray Computer Tomography, we opted for a combined approach. Thirteen landmarks were measured from the left-hand side and midline of the beaks and braincases of 170 psittaciform species housed at the Smithsonian Institution National Museum of Natural History (Figure S1, Tables S5 and S6 in Additional file 4). Landmarks were collected using a MicroScribe G2LX digitiser (Revware Systems, Inc., San Jose, CA), and those from the left were reflected across those from the midline and realigned using FileConverter (http://www.flywings.org.uk/fileConverter_page.htm) to give 20 landmarks in total.

These landmarks were imported in to HyperMesh 11.0 (Altair Engineering Inc., Troy, MI). Surfaces of the beak and skull roof of each specimen were also obtained using a NextEngine laser scanner and MultiDrive running ScanStudio HD Pro 1.3.2 (NextEngine, Inc. Santa Monica, CA), or in the case of two species whose skulls did not fit completely within the field of view of the NextEngine (*Probosciger aterrmius* [from 43 images], *Ara chloroptera* [from 37 images]), with digital photogrammetry (Nikon D3100 DSLR camera, Photoscan 0.9.0, AgiSoft, Russia). These were also imported, unaltered, in to HyperMesh. Because the landmarks and surfaces did not share a common global axis of ordination, the landmarks were translated and rotated to sit atop the surfaces in an appropriate position. Firstly, the landmark configuration was translated so that landmark 1, representing the tip of the beak from the MicroScribed specimen, sat directly on the tip of the beak from the scan. The configuration was then rotated so that the midline (LMs 8, 9, 11, 12, and 13), lateral beak (LMs 2, 3, 14, and 15), and jugal (LMs 6 and 18) landmarks sat in their correct positions on the scan. Semilandmarks were then collected directly in HyperMesh from the dorsal profiles of the beak and braincase, and bilaterally from the tomial edges, then resampled (resample.exe; http://life.bio.sunysb.edu/morph/soft-utility.html) to give 10 equally spaced semilandmarks along each curve. Measurements were performed on specimens without a keratinous rhamphotheca, as these are more commonly preserved in museum collections. All landmarking was performed by one researcher (J.A.B.). While this method probably introduces some error in to the data [41, 42] we expect such error to be consistent across all landmark configurations, and dwarfed by the much larger interspecific differences between skulls. As such, this should not affect the overall pattern of results.

A maximum clade credibility tree for the taxa in the study was constructed using TreeAnnotator [43] (Additional file 4: Figure S2), from a set of 1000 molecular trees with 50% burn-in [44] (www.birdtree.org), and imported to the R environment (v.3.5.1; [45]). The 20 landmarks and 40 semilandmarks were collated for each specimen and imported in to R, where all subsequent morphometric analysis was conducted using the package Geomorph 3.0.7 [46, 47]. The semilandmarks were slid to minimise bending energy [48], then subjected to a Procrustes Superimposition. The symmetric component of shape variation [49] was taken forward to a Principal Components Analysis (PCA) then plotted as a phylomorphospace using the package phytools [50] and tested for phylogenetic signal (Kmult [51]). The skull of a Carolina parakeet (*Conuropsis carolinensis*; NHMUK 1853.7.12.11) was CT scanned (Nikon XT H 225 at the British Museum of Natural History, 0.026 mm resolution, 160 kV, 160 μA) and the bones were segmented and landmarked in Avizo (version 7.0, Visualization Science Group) in order to create visualisations warps (Additional file 4: Figure S1, Additional files 1, 2, 3, and 5).

To get an initial overview of the sources of shape (i.e. the Procrustes aligned landmark configuration) variation in the sample, linear and Procrustes Phylogenetic Generalised Least Squares (PGLS, *procD.pgls* function in Geomorph [52]) regressions were performed against log centroid size (logCS) and diet (shape ~ logCS + diet), with significance assessed over 1000 permutations. Although the difficulties of observing parrots in the wild can hinder detailed knowledge of their diets [18], Wilman et al. [53] have classified Psitticiformes in to three categories of preferred diet which broadly reflect food mechanical resistivity: birds with preferences for generic plant material and seeds; omnivores; and birds with preferences for fruit and nectar. We reinterpret these categories mechanically as birds with preferences for more mechanically resistant (“MMR”; *n* = 72), mixed (*n* = 40), or less mechanically resistant (“LMR”; *n* = 58) food items respectively.

In addition to the initial morphospaces, we generated PCA ordinations of the CS-regression residuals to visualise non size-related (non-allometric) shape changes within Psittaciformes. Covariation between the beak and the braincase was assessed over 1000 permutations using two-block within-configuration phylogenetic PLS [54]. As described in Bright et al. [3], we regressed the PLS1 Block 1 scores of the non-allometric data against those of PLS1 Block 2, then regressed the prediction scores of this regression against the residuals of the regression to log centroid size (the non-allometric shape) using PGLS, the residuals of which were taken to represent the non-allometric, non-integrated shape.

To assess whether shape differences between major clades, and birds with different dietary preferences, were significant, Euclidean pairwise PERMANOVA of the Principal Component (PC) scores across all PCs was conducted using the R package pairwiseAdonis [55]. With the exception of the cockatoos, which have several unifying morphological characteristics [21], the broad similarity of forms within the Psittaciformes has made the classification of groups historically troublesome, and designating clades of equivalent taxonomic rank is not straightforward. Joseph et al. [56] have proposed three superfamilies within Psittaciformes: Strigopoidea (the New Zealand parrots, *Strigops* and *Nestor*; 3 species), Cacatuoidea (cockatoos; 21 species), and the Psittacoidea (true parrots; 374 species). They further divide the Psittacoidea in to three families: the Psittacidae (new world parrots), Psittaculidae (Australasian parrots), and Psittrichasidae (*Psittrichas* and *Coracopsis*). While lories (Loriinae) have traditionally been considered as perhaps warranting family status, they are nested within the Psittaculidae, and should therefore be considered a subfamily. We therefore designated three clades that accommodate these taxonomic difficulties while maintaining analytically useful group sizes: cockatoos (Cacatuidae; *n* = 14), Australasians (Psittaculidae; *n* = 72), and afrotropicals (Psittacidae + Psittrichasidae; *n* = 82). Lories (*n* = 19) were later extracted from the Australasian group and designated as an additional group in post hoc analyses. The Strigopoidea are sister to all other Psittaciformes, and as they were only represented by two specimens in this study, they were excluded from the pairwise analyses as they did not easily fit in to any other monophyletic group. All R code and data associated with our analyses are available in Additional file 6.

## Additional files

Additional file 1: Amination cycling between maximum and minimum warps of PC1, scale factor = 1. Warp template is based on a CT scan of *Conuropsis carolinensis*. (GIF 1082 kb)

Additional file 2: Animation cycling between maximum and minimum warps of PC2, scale factor = 1. Warp template is based on a CT scan of *Conuropsis carolinensis*. (GIF 987 kb)

Additional file 3: Animation cycling between maximum and minimum warps of PC3, scale factor = 1. Warp template is based on a CT scan of *Conuropsis carolinensis*. (GIF 929 kb)

Additional file 4: Supplementary figures and tables. (DOCX 1620 kb)

Additional file 5: Interactive 3D visualisations of the maximum and minimum warps along PCs 1–3. Warp template is based on a CT scan of *Conuropsis carolinensis*. (PDF 33801 kb)

Additional file 6: Zipped folder containing supplementary data files and code. BrightetalParrotsGM.R: R script used for running analysis. R input data: Ccarolinensis.ply; EltonTraits1.csv; landpairs.txt; NewParrots.tre; parrot.lms.txt; Parrot_preSlide.txt; ParrotGroups.csv; sliders.txt. (ZIP 2110 kb)

## Abbreviations

CS: Centroid Size
CT: Computer Tomography
LMR: Less Mechanically Resistant
MMR: More Mechanically Resistant
NANI: Non-allometric Non-integrated
NHMUK: Natural History Museum, UK
PC: Principal Component
PCA: Principal Components Analysis
PGLS: Phylogenetic Generalised Least Squares
PLS: Partial Least Squares
R_PC: post-Regression Principal Component
