# Supplementary figures and images for: The multifactorial nature of beak and skull shape evolution in parrots and cockatoos (Psittaciformes)
Source: BMC Evol Biol. 2019 May 17;19:104. doi: 10.1186/s12862-019-1432-1 (PMC6525378; doi:10.1186/s12862-019-1432-1)

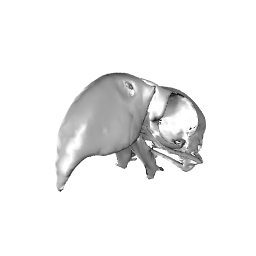

Supplement: Supplementary file 1 — Amination cycling between maximum and minimum warps of PC1, scale factor = 1. Warp template is based on a CT scan of Conuropsis carolinensis. (GIF 1082 kb) [file 12862_2019_1432_MOESM1_ESM.gif]

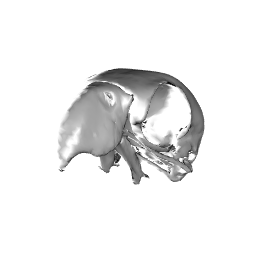

Supplement: Supplementary file 2 — Animation cycling between maximum and minimum warps of PC2, scale factor = 1. Warp template is based on a CT scan of Conuropsis carolinensis. (GIF 987 kb) [file 12862_2019_1432_MOESM2_ESM.gif]

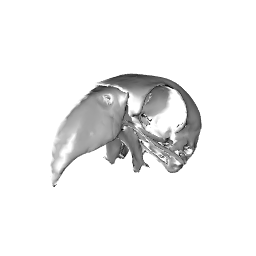

Supplement: Supplementary file 3 — Animation cycling between maximum and minimum warps of PC3, scale factor = 1. Warp template is based on a CT scan of Conuropsis carolinensis. (GIF 929 kb) [file 12862_2019_1432_MOESM3_ESM.gif]
