# Supplementary material for: The multifactorial nature of beak and skull shape evolution in parrots and cockatoos (Psittaciformes)
Source: BMC Evol Biol. 2019 May 17;19:104. doi: 10.1186/s12862-019-1432-1 (PMC6525378; doi:10.1186/s12862-019-1432-1)
Supplement: Supplementary file 4 — Supplementary figures and tables. (DOCX 1620 kb) [file 12862_2019_1432_MOESM4_ESM.docx]

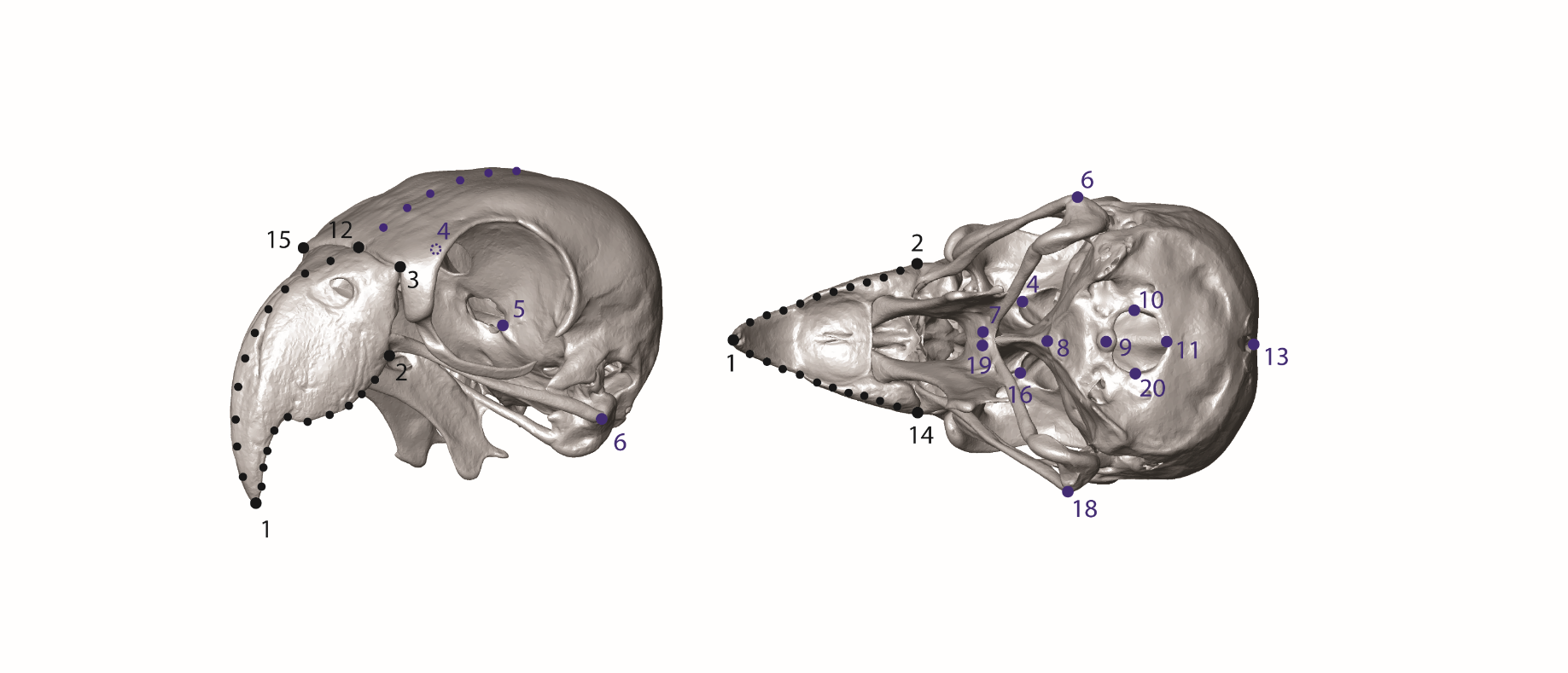
**Fig S1.** Positions of landmarks used in study. Black = beak block, blue = braincase block. Dashed outline of landmark 4 indicates that the exact position of this landmark is obstructed in the oblique view. Species shown is *Conuropsis carolinensis.*

**Fig S2.** Phylogeny used in the study, coloured by clade (pale grey = New Zealand; dark grey = Cockatoos; red = Australasian; orange = Lories; blue = Afrotropical)
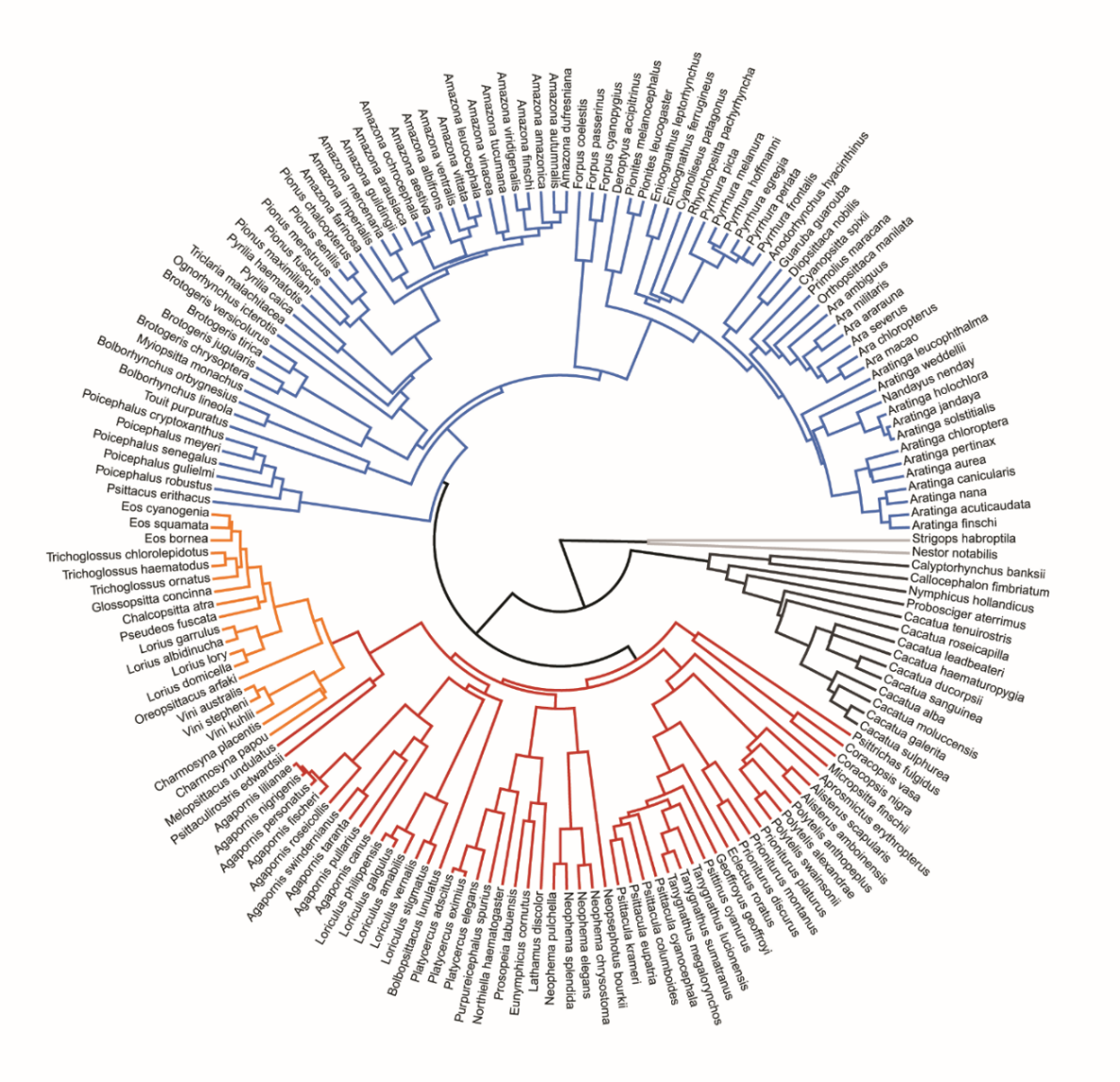


**Table S1**
Diet permanova WITH cockatoos

|  | | | F.Model | R^2^ | p.value | p.adjusted |
| --- | --- | --- | --- | --- | --- | --- |
| LMR | vs | MMR | 3.392672 | 0.02622 | 0.011 | **0.033** |
| LMR | vs | Mixed | 2.336692 | 0.023762 | 0.055 | 0.165 |
| MMR | vs | Mixed | 0.817371 | 0.007511 | 0.475 | 1 |

**Table S2**

Diet permanova WITHOUT cockatoos

|  | | | F.Model | R^2^ | p.value | p.adjusted |
| --- | --- | --- | --- | --- | --- | --- |
| LMR | vs | MMR | 2.803544 | 0.023207 | 0.031 | 0.093 |
| LMR | vs | Mixed | 1.18402 | 0.012985 | 0.269 | 0.807 |
| MMR | vs | Mixed | 1.090535 | 0.011468 | 0.31 | 0.93 |

**Table S3**
Diet in regression residuals (non-allometric) shape, WITH cockatoos

|  | | | F.Model | R^2^ | p.value | p.adjusted |
| --- | --- | --- | --- | --- | --- | --- |
| LMR | vs | MMR | 5.281326 | 0.040229 | 0.002 | ***0.006*** |
| LMR | vs | Mixed | 3.556511 | 0.035724 | 0.015 | **0.045** |
| MMR | vs | Mixed | 0.899297 | 0.008258 | 0.444 | 1 |

**Table S4**
Diet in regression residuals (non-allometric) shape, WITHOUT cockatoos

|  | | | F.Model | R^2^ | p.value | p.adjusted |
| --- | --- | --- | --- | --- | --- | --- |
| LMR | vs | MMR | 3.734926 | 0.030681 | 0.003 | **0.009** |
| LMR | vs | Mixed | 1.219154 | 0.013365 | 0.275 | 0.825 |
| MMR | vs | Mixed | 1.065557 | 0.011209 | 0.334 | 1 |

**Table S5**
Anatomical descriptions of landmark positions. Where landmarks are paired, the lower number represents the landmark on the left and the higher number represents the landmark on the right.

LM 1: Tip of the beak

LM 2, 14: Lateral intersection of the beak with the anterior-most point of the jugal bar

LM 3, 15: Lateral-most projection of the craniofacial hinge midline

LM 4, 16: Anterior/dorsal-most position of the olfactory nerve

LM 5, 17: Posterior/ventral-most position of the orbital nerve

LM 6, 18: Posterior articulation of the jugal bar with the quadrate

LM 7, 19: Articulation between pterygoid and palatine

LM 8: Tube auditiva, midline

LM 9: Centre of occipital condyle

LM 10, 20: Lateral-most point of the foramen magnum

LM 11: Posterior/dorsal-most point of the foramen magnum

LM 12: Midline of the craniofacial hinge

LM 13: Midline crest dorsal to the foramen magnum

Dorsal beak curve = 10 semilandmarks between LM 1 and LM 12

Tomial beak curve (left) = 10 semilandmarks between LM 1 and LM 2

Tomial beak curve (right) = 10 semilandmarks between LM 1 and LM 14

Dorsal skull curve = 10 semilandmarks between LM 12 and LM 13

**Table S6**

Species/specimen list. See ParrotsData.xlsx contained in Additional File 6.
