# Supplementary material for: The multifactorial nature of beak and skull shape evolution in parrots and cockatoos (Psittaciformes)
Source: BMC Evol Biol. 2019 May 17;19:104. doi: 10.1186/s12862-019-1432-1 (PMC6525378; doi:10.1186/s12862-019-1432-1)

The multifactorial nature of beak and skull shape evolution in parrots and cockatoos (Psittaciformes)

Jen A. Bright, Jesús Marugán-Lobón, Emily J. Rayfield, Samuel N. Cobb

BMC Evolutionary Biology

Additional File 5

Interactive 3D visualisations of the positive and negative warps along PCs 1 – 3. Warp template is based on a CT scan of *Conuropsis carolinensis*. Click skulls to activate 3D.

Principal Component 1 (positive)

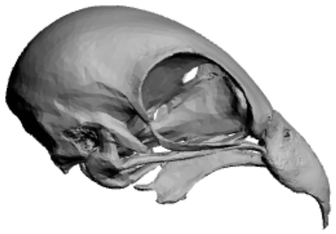

Principal Component 1 (negative)

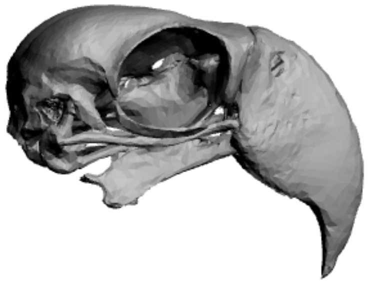

Principal Component 2 (positive)

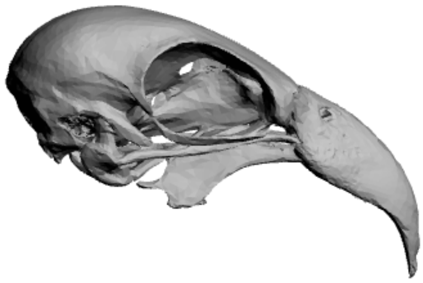

Principal Component 2 (negative)

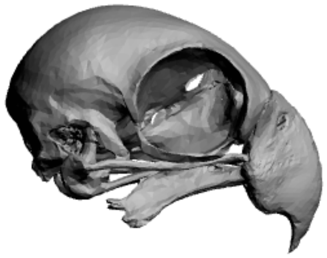

Principal Component 3 (positive)

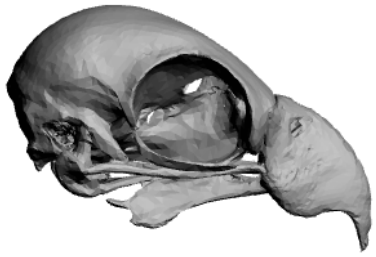

Principal Component 3 (negative)

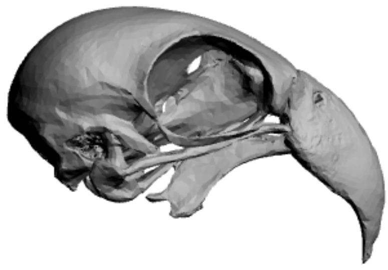

Supplement: Supplementary file 5 — Interactive 3D visualisations of the maximum and minimum warps along PCs 1–3. Warp template is based on a CT scan of Conuropsis carolinensis. (PDF 33801 kb) [file 12862_2019_1432_MOESM5_ESM.pdf]
